# Municipal healthcare providers’ perceptions of reasons for frequent and unplanned hospital admissions among home-dwelling older adults: a Norwegian focus group study

**DOI:** 10.1186/s12877-025-06279-9

**Published:** 2025-08-23

**Authors:** Jill-Marit Moholt, Cathrine Arntzen, Audhild Høyem, Bodil H. Blix

**Affiliations:** 1 Centre for Care Research North, Department of Health and Care Sciences, Faculty of Health Sciences, UiT The Arctic University of Norway, Tromsø, Norway; 2 Department of Rehabilitation Services, University Hospital of North Norway, Tromsø, Norway; 3 Department of Collaboration, Innovation and Health Service Development, University Hospital of North Norway, Tromsø, Norway; 4 Department of Health and Care Sciences, Faculty of Health Sciences, UiT The Arctic University of Norway, Tromsø, Norway; 5 Department of Arts Education, Faculty of Education, Arts and Sports, Western Norway University of Applied Sciences, Bergen, Norway

**Keywords:** Emergency admissions, Focus group, Frequent users, Hospital admissions, Hospital services, Integrated care, Municipal healthcare services, Older adults, Primary care, Qualitative study

## Abstract

**Background:**

In healthcare policies and research, patients who utilise substantial healthcare services receive significant attention. Persons who are frequently admitted to hospitals are often termed ‘frequent users of hospital services’. Understanding how front-line healthcare providers in municipal healthcare services perceive these patients’ healthcare needs is crucial for developing adequate and integrated care across healthcare levels. This study aimed to examine how healthcare providers within municipal healthcare services recognise and describe the healthcare needs of home-dwelling older adults with frequent and unplanned admissions to somatic hospitals. A central aspect of the study was to explore what healthcare providers identify as the reasons for these hospital admissions.

**Methods:**

This qualitative study employed an exploratory design. We conducted six focus group discussions involving a total of 26 healthcare providers from home-based services and institutional short-term wards in four Norwegian municipalities. The data were analysed using a six-step reflexive thematic analysis.

**Results:**

The participants’ perceptions of the needs and reasons for unplanned hospital admissions among home-dwelling older frequent service users included aspects at both the individual and structural levels. At the individual level, healthcare providers highlighted patient characteristics such as the severity of illness, reduced functional ability, living situation, and unmet psychosocial needs. At the structural level, participants emphasised system issues that could intensify the need for hospital admissions, such as in-hospital care failing to address complexity before discharge, hasty discharges with insufficient information sharing, and a lack of essential and comprehensive municipal healthcare services, such as home-based night care.

**Conclusions:**

This study demonstrates that healthcare needs and reasons for unplanned hospital admissions among older, frequent users are multifaceted and influenced by both individual and structural factors. Based on our findings, we recommend that healthcare policies aimed at reducing unplanned and potentially avoidable hospital admissions address structural-level issues. This includes ensuring access to essential and comprehensive municipal healthcare services and fostering integrated care through collaboration and dialogue between hospitals and municipal healthcare services.

**Supplementary Information:**

The online version contains supplementary material available at 10.1186/s12877-025-06279-9.

## Background

In recent decades, there has been increased attention on patients who extensively use healthcare resources, both in terms of costs and the frequency of service use [1, 2]. Patients who frequently utilise healthcare services are often referred to as ‘frequent users’ [3–5]. Previous research indicates that these patients constitute a heterogeneous group with diverse healthcare needs that require tailored treatment and care approaches [4–8]. In this study, we concentrated on home-dwelling older adults with frequent and unplanned admissions to somatic hospitals.

Unplanned hospital admissions are those where patients are admitted to the hospital urgently and unexpectedly (i.e., emergency admissions) due to acute or worsening long-term health conditions and trauma [9]. While many of these admissions are unavoidable, health policies emphasise reducing potentially *avoidable* hospitalisation [1, 2, 10, 11]. This is considered important for alleviating the burden on patients, reducing costs and achieving better resource utilisation in hospitals [2, 9]. OECD reports [2, 11, 12] particularly highlight avoidable hospital admissions among patients with chronic health conditions such as asthma, diabetes, chronic obstructive pulmonary disease (COPD), and congestive heart failure, noting that “the evidence base for effective treatment is well established, and much of it can be delivered by primary care” ([12] p. 132). Additionally, significant healthcare policy attention is given to unplanned and *avoidable readmissions*, which are potentially preventable readmissions occurring shortly after hospital discharge [13, 14]. Although this study did not specifically address readmissions, they represent a type of unplanned hospital admission that contributes to increased service use and the frequency of hospitalisations.

Previous research concerning frequent users of emergency departments and hospital services suggests that these patients may suffer from various medical and chronic conditions [7, 8]. Additionally, they might experience unmet healthcare needs, insufficient support for activities of daily living (ADLs), and unmet social needs [3, 15]. These unmet needs may place patients at risk for deterioration of health status and, ultimately, lead to unplanned admissions to hospitals [16]. Research also indicates that reasons for frequent and unplanned hospital admissions, including readmissions, are influenced by factors within both specialised and primary healthcare services, as well as cross-sectorial conditions. These factors include premature discharge from hospitals [17] and insufficient transitional care, such as inadequate information sharing and coordination of follow-up care [15]. Furthermore, inadequate patient follow-up from primary care [15, 17] and barriers to accessing primary healthcare [16–18] are other factors that might contribute to hospitalisation.

While numerous studies have examined frequent users of emergency departments or hospital services among adult populations, fewer have specifically addressed older adults [4]. Even fewer studies have explored the perspectives of municipal healthcare providers regarding older frequent users, despite healthcare policies emphasising their crucial role in assessing and meeting older populations’ healthcare needs [1], as well as ensuring continuity of care and implementing integrated care strategies [19]. Qualitative research involving healthcare providers can offer valuable in-depth insights into the complex relationships among the healthcare needs of older patients, service provision, and the reasons behind their frequent use of services. This knowledge is essential for developing interventions and improvement strategies aimed at enhancing the quality of patient care, ultimately helping to reduce unplanned and potentially avoidable hospital admissions [1].

Based on these considerations, the aim of this study was to examine how healthcare providers within municipal healthcare services recognise and describe healthcare needs and reasons for hospital admissions among home-dwelling older adults with frequent and unplanned admissions to somatic hospitals. The research questions were as follows: How do municipal healthcare providers perceive the healthcare needs of older adults who are frequently admitted to hospitals, and what do they identify as the reasons for unplanned hospital admissions?

Furthermore, we alternate the use of the terms (older) frequent users, patients, or care recipients when referring to the older adults in this study.

## Methods

This study is part of a broader multi-method research initiative that concerns older adults who utilise substantial resources in Norwegian somatic hospitals in terms of both the costs and frequency of service use, as well as patients’ use of municipal healthcare services. This particular study uses an exploratory design, which is based on focus group discussions with healthcare providers from municipal healthcare services. Throughout the development of the project, a reference group actively provided feedback and offered advice to the research team. This group included patient and family representatives, healthcare providers from both municipal and specialised healthcare services, and researchers. In this study, the reference group provided feedback on themes and questions in the interview guide. Additionally, we discussed preliminary results with the group, and both healthcare professionals and user representatives confirmed that the findings were relevant and recognisable.

### Study setting

The Norwegian public healthcare system is divided into specialist and primary services. The specialist services, including somatic hospitals, are managed by the state and are operated by four regional health authorities. Primary healthcare services, hereafter referred to as municipal healthcare services, are run by the municipalities [20]. Municipal healthcare services encompass statutory services, including general practitioners, municipal emergency centres, municipal rehabilitation services, home-based services, and institutional care in nursing homes, which may involve either short- or long-term care [21]. In accordance with national healthcare policies, municipal healthcare services at lower care levels, like home-based services, should be utilised before care recipients are allocated more resource-intensive services at higher care levels, such as institutional care in nursing homes [22].

While hospital healthcare services are oriented towards diagnoses and targeted medical treatments, municipal healthcare services such as home-based and institutional care are centred on patients’ functioning and coping, along with providing comprehensive long-term care [23]. In recent years, healthcare authorities have launched reforms and strategies concerning the organisational aspects of services as well as the integration of care between the two healthcare sectors [20]. Following the Coordination Reform in 2012 [24], municipalities were given expanded responsibility to address the healthcare needs of citizens. At the same time, measures were implemented to ensure more appropriate use of hospital services and to increase the hospitals’ capacity for highly specialised treatments. For instance, municipalities were given the main responsibility for patients who are ready to be discharged and must pay a daily fee to the hospital for patients who remain there while waiting for municipal healthcare services [20, 24]. Moreover, municipal inpatient acute care services were established with the goal of reducing the need for hospital admissions [24, 25]. Despite these initiatives, research conducted after the implementation of the reform indicated that significant challenges persist regarding insufficient collaboration and fragmented care pathways between the two healthcare sectors [26, 27].

### Recruitment and participants

At the time of data collection (2020–2021), there were 356 municipalities in Norway [28]. We recruited participants from four municipalities, which were strategically selected to obtain variation in demographic characteristics such as centrality, number of inhabitants and distance to the nearest hospital.

We employed a strategic sampling approach to select healthcare providers involved in care of home-dwelling older adults within municipal healthcare services. In this study, this included experienced healthcare providers from municipal home-based services, rehabilitation services, or short-term wards within nursing homes. The rationale for including participants from the latter category was their role in providing care to home-dwelling patients requiring institutional care for a limited period, such as short-term care or rehabilitation following hospital discharge.

Four out of five invited chief managers consented on behalf of their municipalities to participate in the study and assist in the recruitment of participants. Approximately 40 healthcare providers received written information about the study, 30 of whom provided written consent to participate. In total, four healthcare providers withdrew their consent or were unable to attend the scheduled focus group discussions. We did not ask the invited providers for their reasons for nonparticipation. Nonetheless, it is noteworthy that the study took place during the COVID-19 pandemic, and some managers indicated that sick leave, workload, and pandemic-related duties, including the vaccination of patients, hampered providers’ ability to participate.

Details on the included municipalities, as well as the number of focus groups and participants in each group, are presented in Table 1. An overview of the focus groups and the participants is provided in Supplementary File 1. All 26 participants were women, and they had an average of 15 years of experience (ranging from 2 to 43 years) in municipal healthcare services. At the time of the focus group discussion, three of the participants held positions as middle managers in a day care centre, short-term ward, or rehabilitation unit. Despite their managerial role, all had recent experiences in clinical practice, and they were encouraged to draw upon these experiences during the discussions.

**Table 1:** Details on the municipalities and focus groups included in the study

	Centrality*	Number of inhabitants	Distance to nearest hospital	Focus group (FG)	Number and designation of healthcare provides (HCP)
Municipality 1	Least central municipality	~ 5,000	> 100 km	FG 1	5 (HCP 1–1– HCP 1–5)
Municipality 1				FG 2	6 (HCP 2−1– HCP 2–6)
Municipality 2	Second least central municipality	~ 10, 000	Within the municipality	FG 3	2 (HCP 3−1– HCP 3−2)
Municipality 2				FG 4	2 (HCP 4−1– HCP 4−2)
Municipality 3	Mid-central municipality; small town	~ 20, 000	> 100 km	FG 5	6 (HCP 5−1– HCP 5–6)
Municipality 4	Mid-central municipality; city	> 50, 000	Within the municipality	FG 6	5 (HCP 6−1– HCP 6−5)

*FG* focus group, *HCP *healthcare provider. HCP 1–1 indicates that the participant was in the first focus group and is designated as the first participant, etc

*The classification is based on Statistics Norway’s index of municipal centrality, which calculates centrality using municipal characteristics such as distance to urban centres, share of the population living in urban areas, and distance to workplaces and services [29]

### Data generation

We considered focus group discussions an appropriate data collection approach, as the interactive format allows for different perspectives and opinions, and it is an effective method for creating a large and rich dataset [30]. We conducted a total of six focus group discussions, each consisting of two to six participants, from October 2020 to March 2021. Focus group discussions one, two, and six were conducted face-to-face at the participants’ workplace, whereas discussions three to five were conducted on the digital platform Teams due to a new wave of the COVID-19 pandemic and the subsequent strengthening of infection control measures. Focus groups three and four were originally planned as a single group. However, it was difficult to schedule a convenient meeting time due to changes in shift schedules and pandemic-related tasks that had to be prioritised. After several attempts, we decided to arrange two separate discussions with two participants each, which is fewer than the literature’s recommended group size of at least four or five participants [30, 31].

We developed a semi-structured interview guide based on the overarching objectives of our study, previous research in the field (e.g., [4, 5, 7, 32, 33]) and the research team’s expertise. Additionally, we incorporated feedback from the reference group. The interview guide is presented in Supplementary File 2. The participants engaged in discussions on various topics, including the characteristics and healthcare needs of older frequent users, strategies for preventing hospital admissions, and collaboration with healthcare providers in hospital services. There was a potential risk of participants disclosing personal data about third parties such as patients and healthcare personnel. To mitigate this risk, the moderator began each focus group discussion with a reminder to participants not to share identifiable personal information such as names and places of residence. This precaution was maintained throughout all discussions.

AH was the moderator in the first three focus group discussions, while JMM acted as the co-moderator. In the last three discussions, we alternated roles. The role of the moderator was to lead the interviews, which included starting the sessions with information about the study, asking questions, creating space for discussions among all participants, and summarising the discussion at the end. The co-moderator’s role was to observe the participants and their interactions, as well as to take notes related to group dynamics and nonverbal communication [30]. Additionally, the co-moderator asked follow-up questions or assisted the moderator if needed. Each focus group discussion lasted approximately 50 minutes and was audio recorded and transcribed verbatim by JMM and AH. The transcriptions were anonymized by removing the names of participants, municipalities, and hospitals.

### Analysis

The data were analysed according to the principles of reflexive thematic analysis [34]. In this study, we employed an inductive approach driven by the data content. The analysis process consisted of six phases [34]. In the first phase, i.e., *familiarisation*, JMM and AH read the transcribed interviews several times, noted interesting aspects, and discussed their initial impressions of the data. In the second phase, *systematic data coding*, JMM assigned code labels to the data and collated data segments related to each code. In the third phase, *initial theme generation*, JMM developed collated codes into potential themes by identifying meaningful patterns in the data. The fourth phase involved *reviewing and developing themes*, which entailed re-examining the list of codes and refining the collated data. In the latter two steps, JMM involved BHB to discuss the development of the themes. During the fifth phase, which involved *refining*,* defining*,* and naming the themes*, the preliminary themes and subthemes were presented to all the coauthors. The themes were evaluated and refined until a consensus was reached. The sixth phase involved *producing the report*. During this process, JMM prepared an initial draft of the results section of this article and presented it to the author group. JMM and BHB worked together to refine it, and all coauthors provided feedback and participated in the subsequent writing process until the final version was completed.

All researchers involved in this study have professional backgrounds in healthcare, including nursing and occupational therapy. We all have clinical experience from specialists and/or municipal healthcare services, but none of us are currently working in patient-oriented work. Additionally, we hold a PhD in health science and have specialised expertise in gerontology and health services research. Our background informed our assumptions and expectations regarding the healthcare needs of older patients in this study and the reasons for their hospital admissions. As a reflexivity strategy, the focus group moderators discussed their experiences from clinical work and previous research before conducting the focus group discussions. For example, we anticipated that participants would emphasise complex and long-term health conditions when describing the healthcare needs of the patients in question, as well as collaboration challenges associated with the transition of care from hospitals to municipal services. After each interview, we critically discussed our role as researchers and how our engagement influenced the discussions. We were also conscious of how our professional backgrounds and experiences could affect the analysis. By involving members of the research team in various phases of the analysis, a practice known as investigator triangulation, we aimed to reduce the risk of biased interpretations and decisions [30].

## Results

When we asked participants about older patients with frequent and unplanned admissions to hospitals, they described these patients as having a variety of complex and long-term healthcare needs. Additionally, they expressed that these patients often utilised multiple municipal healthcare services, such as general practitioners, home-based services, short-term institutional care, rehabilitation services, or activity services such as day care centres. Further analysis revealed that participants’ perceptions of the healthcare needs of these patients and the reasons for their unplanned hospital admissions included both individual- and structural-level aspects. This insight led to the development of the two main themes: (1) Individual-level aspects related to frequent and unplanned hospital admissions, and (2) Structural-level aspects related to frequent and unplanned hospital admissions. The themes and related sub-themes are presented in Table 2.

**Table 2:** Overview of main themes and related sub-themes

Main themes	Sub-themes
Individual-level aspects related to frequent and unplanned hospital admissions	Severe illness necessitates the use of multiple healthcare services Reduced functional abilities are part of a complex pattern of healthcare needs Living situation and unmet psychosocial needs impact overall healthcare needs
Structural-level aspects related to frequent and unplanned hospital admissions	In-hospital care fails to address complexity before discharge Hasty hospital discharges and insufficient information Lack of essential and comprehensive municipal healthcare services

### Individual-level aspects related to frequent and unplanned hospital admissions

The results show that participants identified several individual-level aspects contributing to frequent and unplanned hospital admissions. These aspects were organised into three subthemes, primarily linked to older patients’ illnesses, functional abilities, living situations, and unmet psychosocial needs.

#### Severe illness necessitates the use of multiple healthcare services

Participants agreed that older adults with severe chronic and incurable illnesses were predisposed to frequent and unplanned hospital admissions. Although the participants mentioned several illnesses that they associated with the need for in-hospital care, they particularly emphasised patients with chronic obstructive pulmonary disease (COPD), chronic heart and kidney failure, and incurable or terminal cancer. According to the participants, deterioration of health status and severe symptoms could result in multiple and unplanned hospital admissions, especially in advanced or terminal phases of the illness when symptoms occurred frequently or were almost continuously present. Additionally, acute illnesses such as infections could lead to worsening of symptoms of the underlying illness(es), resulting in the need for urgent hospital treatment. Several of these conditions were highlighted at the beginning of one of the group discussions, where a nurse from the home-based services engaged the oncology nurse in sharing her experiences:

*HCP 1–1: Often*,* older patients have complex conditions*,* such as heart failure and COPD. And those who have infections. Those who suddenly become ill and are severely affected by infections* […]. *Yes*,* I’ll say that patients with heart failure and COPD are those who’re most in and out of hospitals. [The rest of the participants agreed by nodding and saying “yes”]. However*,* perhaps you can say something about the patients with cancer? [Turns to the oncology nurse].*

*HCP 1–3: Yes*,* as the disease progresses*,* patients can be frequently admitted due to fluid therapy or tapping of fluid*,* infections*,* pain*,* [need of] observations*,* and clinical examinations.*

Some participants expressed that the hospitals sometimes lacked additional treatment options, particularly in the advanced stages of patients’ chronic or incurable illnesses. They perceived that while in-hospital care temporarily improved patients’ conditions, their health status could rapidly deteriorate after discharge. This pattern often led to what some of the participants described as “roundtrips”, where patients were readmitted to the hospital after a short period at home. In one group, two nurses in home-based services specifically highlighted this issue in relation to older adults with severe COPD:

*HCP 6−5: It reaches a point where it’s no longer possible [to stay at home]. [The patients] are admitted [to hospitals] to receive treatment*,* and afterwards*,* they come home again.*

*HCP 6−4: In several cases*,* there is no treatment to offer. That’s what happens. Then*,* the patients come home*,* and their health condition may be the same. [The participants agree and speak simultaneously about similar experiences].*

The engagement in the group and the subsequent discussion indicated that this was a well-known concern related to older adults with COPD, as well as for patients with other severe illnesses. The participants further explained that transferring these older patients to intermediate care within the municipality was an option if their health conditions worsened, but, as in the hospital, it was challenging to provide sufficient treatment. A nurse in home-based services explained:

*HCP 6−5: [The patients] are admitted to the municipal inpatient acute care unit*,* where they can stay [for a few days]. However*,* then there’s no treatment to offer them*,* so they come home again. Then*,* they are home for a day*,* and then we send them [to the hospital]. It is very unfortunate.*

At the same time, the participants noted that these patients receive much assistance from home-based services. A nurse claimed: *“We are maximally involved with many of them” (HCP 6–4).* Some participants described initiating treatments at home, including administering prescribed medications for patients with severe symptoms. Such local measures were sometimes adequate to prevent hospital admissions. A nurse in home-based services in the smallest municipality highlighted the importance of being familiar with patients and understanding their needs, which enabled timely interventions such as transferring patients to an appropriate level of care:

*HCP 1–4: I think there is a need when they’re admitted [to the hospital]. I truly believe so. Because the positive thing is that we often know them very well. In some cases*,* we can admit them for a stay [to the municipal inpatient acute care unit or the short-term ward].*

Overall, these findings indicate that many older adults with severe illnesses and complex healthcare needs experience frequent transfers, not only between their home and the hospital but also between different healthcare levels within the municipality.

#### Reduced functional abilities are part of a complex pattern of healthcare needs

Some participants highlighted that older adults with complex and long-term conditions are now living in their own homes for longer periods compared to a few years ago. They expressed particular concern about patients of advanced age who have reduced functional ability, experience unsteadiness, and tend to fall, which can be exacerbated by underlying conditions and illnesses. Several of these aspects, along with acute cognitive decline, were emphasised in a discussion between two nurses in home-based services:

*HCP 1–4: However*,* several patients have multimorbidity and are older persons who continue to live at home. We notice that more and more. The whole picture is more complex. Fall tendencies are something we are very concerned about and try to prevent. [Several participants confirmed by saying “yes”].*

*HCP 1–1: Acute cognitive impairment and…*.

*HCP 1–4:… Yes*,* a lot of that.*

Some participants expressed that patients’ functional abilities could be worsened by acute illnesses such as infections, poor nutritional status, or medications. In this context, they emphasised falls and hip fractures as reasons for hospital admissions:

*HCP 5−1: [The home-dwelling older patients] often have functional decline and perhaps an infection. The situation subsequently escalates with a fall at home*,* which leads to further functional decline. However*,* infections or hip fractures often recur [as reasons for hospital admissions].*

*HCP 5−3: Yes*,* we have the same experiences [in the short-term ward]. Additionally*,* they often have functional decline and nutritional deficiencies that make them more ill. There are also issues with medication […].*

Although the participants highlighted falls and hip fractures, these conditions might not necessarily lead to frequent hospitalisations on their own. As indicated in the quote above (HCP 5−1), such incidents could exacerbate the overall needs of older patients who already have complex and long-term healthcare needs. This, in turn, could make them more disposed to hospitalisation. Moreover, some participants questioned whether older adults of advanced age with such substantial needs should continue to live at home.

#### Living situation and unmet psychosocial needs impact overall healthcare needs

The participants perceived that living with someone or having family nearby served as protection against unplanned hospital admissions. Family members could provide care, monitor changes in health conditions, and contact municipal healthcare services, such as general practitioners or home-based services, when needed. In contrast, several participants specifically noted the vulnerability of older care recipients living alone without nearby family support. An occupational therapist emphasised the link between patients’ complex healthcare needs, family caregiving, and frequent hospital admissions:

*HCP 3−2: It’s often those who live alone who are most frequently admitted to the hospital. Yes. However*,* it’s also because they have [chronic illnesses] and functional decline […]. Those who live with a family member have someone present who can act quickly if things worsen. They can monitor the care recipient at home throughout the day and night […].*

Some participants highlighted that older care recipients with complex healthcare needs who lived alone could feel lonely, anxious, or unsafe at home despite frequent visits from home-based services. The following quote illustrates how unmet psychosocial needs were associated with a lower threshold for hospital admissions:

*HCP 4−2: Several [older adults] are living alone and have poor social networks. Their family might live far away. Except for [healthcare providers in home-based services]*,* they don’t have anyone visiting them daily. They’re feeling very lonely […]. I think that anxiety is an additional issue […].*

*HCP 4−1: Yes*,* it is easier when you have a [social] network*,* then you’re safer. You’re tolerating more. Tolerating to be at home. The path to [hospital] admissions is shorter when you’re anxious and… yes.*

Some participants also expressed that care recipients of advanced age were reluctant to participate in social activities in the community. As one nurse in home-based services noted:

*HCP 4−2: I believe it’s the old age that is the reason [not to participate in social activities outside their home]. They have been active before and then they get older and become increasingly confined in their own home […].*

It is conceivable that isolation at home could, in turn, increase care recipients’ unmet psychosocial needs, potentially increasing the complexity of their overall healthcare needs.

### Structural-level aspects related to frequent and unplanned hospital admissions

When discussing healthcare needs and the reasons for frequent and unplanned hospital admissions among patients, several participants emphasised aspects beyond individual patient characteristics. These primarily involved structural-level conditions related to hospital and municipal healthcare services. We organised the results into three subthemes, as shown in Table 2.

#### In-hospital care fails to address complexity before discharge

A common understanding among the participants was that the duration of hospital stays has decreased in recent years, resulting in patients being discharged earlier than previously. Consequently, they perceived that care recipients have higher healthcare needs upon their return to the municipalities than they did a few years ago, leading some to refer to them as “another type” of patients within the services. Two experienced nurses in home-based services reflected on this issue:

*HCP 1–4: I’m thinking*,* they [the patients] are more ill now than they were previously. I’m seeing that*.

*HCP 1–3: However*,* this is obvious since [the patients] are discharged earlier.*

*HCP 1–4: Yes*,* much earlier. There is another type of patient*,* perhaps.*

Some participants indicated that the short hospital stays reflected a “non-holistic” care approach, meaning that in-hospital care centred on the specific diagnosis or condition that caused admission, such as a hip fracture, rather than on the complex healthcare needs of the patients. According to the participants, this resulted in discharge as soon as the specific illness or injury was treated, such as repositioning of the hip fracture. A nurse in home-based services claimed:

*HCP 5−1: Patients’ healthcare needs are too complex and time consuming to address during the [short] hospital stay. Sometimes*,* I ask myself if each treatment is paid individually*,* so that the hospital just fixes one thing at time. […]. The patients must be discharged as soon as possible.*

Some participants believed that a holistic assessment of older patients’ complex healthcare needs, including mapping of underlying illnesses and care needs, was crucial for determining whether patients could be discharged from hospitals to municipal services. Their impression was that such assessments were rarely performed before discharge, and thus, it was not recognised that patients could be too ill to be followed up by municipal healthcare services. Consequently, discharge can be followed by rapid readmission to the hospital. A physiotherapist working in a rehabilitation unit highlighted this issue:

*HCP 5–6: We*,* who’re working in physiotherapy and occupational therapy services*,* encounter several of the patients who are frequently admitted to hospitals […]. The clinical assessment of patients is not complete before they are discharged. Therefore*,* they’re simply too sick to stay in [the short-term ward] or at home*,* and thus*,* they’re readmitted to the hospital*.

This suggests that short hospital stays, where patients’ overall condition is not sufficiently assessed before discharge, may not actually reduce the use of hospital resources, as this can result in patients being readmitted.

#### Hasty hospital discharges and insufficient information

A related topic was the issue of hasty hospital discharges of patients. According to the participants, these discharges often resulted in patients being returned to the municipalities without the necessary information, technical aids, medical equipment, and medical prescriptions. They considered this situation disadvantageous for patients because neither their homes nor follow-up initiatives by municipal healthcare services were adequately prepared prior to discharge. Additionally, participants frequently encountered situations where discharge summaries detailing in-hospital treatment and medication were not completed and sent to municipal healthcare services at the time of discharge. This posed challenges for municipal healthcare providers, as such information was crucial for patient follow-up. The following discussion between nurses in home-based services illustrates concerns about the increasing frequency of hasty discharges and insufficient information:

*HCP 6−2: [Hasty discharges] have happened several times lately. [The rest of the participants agreed].*

*HCP 6−4: Discharge summaries and medication lists are missing because they have not been signed by the physician.*

*HCP 6−1: Consequently*,* patients come home without medical prescription*,* and nothing has been arranged for them.*

Hasty hospital discharges were seen as particularly problematic when the patients were sent home on Friday afternoons, which, according to several participants, had become more frequent. One focus group referred to this situation as the “Friday bottleneck”. The Friday bottleneck was characterised by reduced staff availability as the weekend approached combined with challenges with securing necessary equipment and medications due to closed support services such as the pharmacy. In one discussion, participants immediately highlighted these challenges when they discussed the healthcare needs of older frequent users, suggesting that this was particularly crucial to understanding frequent hospital admissions:

*HCP 5−1: We often see that the patients come home too early. Therefore*,* it can lead to round trips [readmission to the hospital]*,* which are often unnecessary. […] The patients are coming home late on Fridays. Their home isn’t prepared. There is no technical aid in place. They’re coming home without medical prescriptions or equipment. In addition*,* then*,* we in the home-based services must deal with it. […] And in several cases*,* unfortunately for the patients*,* it’s back to hospital again. This is unworthy and a great strain to them.*

*HCP 5–6: It’s like [HCP 5−1] is saying; we’re often given short time frames. It’s almost like: “Now*,* the patient is in the ambulance on his way to [the municipality]*,* you must manage that”. And we don’t always manage*.

Additionally, this suggests that participants perceived that hasty discharges and readmissions to hospitals (cf. “round trips”) were demanding not only for older patients but also for municipal healthcare providers, who were not always able to provide sufficient healthcare to patients.

#### Lack of essential and comprehensive municipal healthcare services

In some discussions, participants highlighted the lack of essential and comprehensive municipal healthcare services, which they perceived as contributing to hospital admissions among patients. Although the municipalities differed in size and number of inhabitants, all included large rural and remote areas. Healthcare services are often located in municipality centres, which, according to the participants, could affect accessibility for those living in remote areas. For example, healthcare providers in home-based services could not visit care recipients in remote areas several times a day or in evenings due to long travel distances:

*HCP 2–2: […] If [care recipients] live 20–30 km away [from the community centre] and need help in the evening*,* we have experienced that we couldn’t visit the patient within the shift.*

Additionally, one municipality did not offer home-based night services, whereas others were unable to provide night care to patients who lived outside the community center. According to the participants, low accessibility or lack of round-the-clock home-based services could affect the provision of care and follow-up of patients, including those recently discharged from the hospital. This also presented dilemmas for the healthcare providers, as the patients in need of assistance had to manage on their own for large parts of the day. This issue was apparent when nurses from the municipality without home-based night care discussed the situation:

*HCP 1–1: When something happens in the evening*,* we should observe the patient during the coming night. However*,* we go home and know nothing…*.

*HCP 1–3:…until you return next morning.*

*HCP 1–1: You’re just hoping things are going well until you’re back the next morning.*

*HCP 1–4: Yes*,* that’s how it is.*

If the health condition of patients deteriorated during the night, they required assistance from other healthcare services, potentially leading to hospital admissions:

*HCP 1–1: In some cases*,* the ambulance has brought patients to the hospital at night.*

*HCP 1–2: Or to the nursing home. However*,* there are often no places available there*.

The above quote indicates limited availability of short-term care in nursing homes. This was further confirmed in other discussions, where the participants highlighted issues such as exceeded capacity and scarcity of available places. Additionally, some participants expressed concerns regarding waiting lists for long-term nursing home care, believing that several of the patients in question were too ill to manage at home given their extensive healthcare needs. A nurse in home-based services highlighted the risk of acute incidents at home, which potentially could lead to hospital admissions:

*HCP 5−1: In fact*,* it is unrealistic that [the patients] should live alone at home. In several cases*,* this is irresponsible. We’re almost waiting for falls and bone fractures. Yes*,* I often think this is irresponsible.*

The connection between the lack of long-term care places in nursing homes and hospital admissions was also emphasised in a discussion between nurses in home-based services about the healthcare needs of older patients with severe COPD in an advanced stage of their disease:

*HCP 6−5: Usually*,* they’re dying while they are in the queue [on the waiting list for long-term care in the nursing home].*

*HCP 6–6: At the end of life*,* patients are more frequently admitted to hospitals. Yes.*

Altogether, these findings demonstrate structural challenges in providing municipal healthcare services at an appropriate level that correspond to the complex healthcare needs of older frequent users.

## Discussion

The participants in this study perceived healthcare needs and reasons for hospital admissions among older, frequent users as multifaceted and shaped by both individual and structural factors. In the following sections, we discuss our results in the context of key health policy strategies aimed at strengthening the role of municipal healthcare services in providing care for patients with complex and long-term needs, including those with frequent and unplanned admissions to somatic hospitals [10, 12, 19, 24].

Healthcare policies emphasise the critical role of primary care in reducing unplanned and potentially avoidable hospital admissions by effectively managing long-term conditions such as heart failure and COPD, and initiating early interventions in cases of acute exacerbation of these illnesses [12]. Consequently, hospital admission rates for these conditions serve as international indicators of the quality and access to primary care [10, 12]. Heart failure and COPD were also conditions emphasised by the participants in our study when describing the healthcare needs of older frequent users. Other individual-level aspects, not captured by admission rates but highlighted by the participants, included the presence of comorbidities and acute illnesses such as infections, functional decline and fall risks, living situation, unmet psychosocial needs, and end-of-life care. A Norwegian cohort study of hospital admissions among home-dwelling older adults receiving municipal healthcare services reported similar reasons for hospitalization, such as general decline, infections and falls [35]. A national register study indicated that older adults with three or more main diagnoses and those who passed away during the study period were more likely to use substantial hospital resources [36]. Similarly, a Swedish cohort study demonstrated that unplanned hospital admissions are common in the last month of life [37]. Overall, the results of our study, along with those of previous studies, demonstrate that a range of individual factors should be considered when assessing hospitalisation rates and determining whether unplanned hospital admissions are avoidable.

In this study, several participants emphasised unmet psychosocial needs such as anxiety, feelings of insecurity, loneliness, and social isolation, when describing the healthcare needs of older frequent users. There is substantial evidence indicating that unmet social and emotional needs are risk factors for serious health outcomes such as cognitive decline and depression, but little is known about how these needs impact healthcare utilisation [38]. Our results suggest that these individual-level aspects add to an already complex profile of healthcare needs, potentially intensifying the demand for healthcare services, including the need for hospitalisation. These relationships should be addressed more thoroughly in future studies.

Integrated care across healthcare services and sectors is crucial for preventing adverse health outcomes, unmet healthcare needs, and extensive use of healthcare services among older adults with chronic and complex conditions [10, 12, 19, 24]. Notably, our data collection was carried out nearly a decade after the implementation of the Norwegian Coordination Reform, which emphasised necessary prerequisites for integrated patient care, such as interprofessional collaboration and information sharing across healthcare sectors [24]. Our study indicates that significant structural challenges persist in the transition of care from hospitals to municipal healthcare services. Among these challenges are in-hospital care that fails to assess and address patients’ overall healthcare needs before discharge, and hasty discharges with inadequate information exchange. This can lead to readmissions, many of which could have been avoided with better integration of care. A previous Swedish focus group study [39] among hospital physicians indicated that hospital care is not adequately tailored to older patients with multimorbidity. The physicians reported a lack of competence in geriatrics and insufficient time to adequately address the complex needs of the patients. Additionally, they described high turnover of staff and a shortage of beds, which could lead to a lack of continuity of care and hasty, premature discharges. Similar findings have been reported in interview studies with hospital physicians about older patients’ care pathways between hospital and municipal healthcare services [40] and readmission processes [41].

Hasty discharges were also emphasised by the participants in our study, which were particularly challenging when patients were unexpectedly discharged at the end of the week (cf. “the Friday bottleneck”). Research has demonstrated that readmissions occur more frequently when patients are discharged on Fridays [42, 43]. This is explained by fewer staff on duty in hospital wards over the weekend, resulting in rushed and insufficient discharge procedures [42]. Our study shows that there are also fewer staff at work in municipal healthcare services, making it challenging to provide care to recently discharged patients due to a lack of equipment, medications, and necessary information essential for care planning, such as discharge summaries. Moreover, these shortcomings could be even more problematic in rural areas due to long travel distances from the location of services to patients’ homes, as well as the lack of pharmacies or their limited opening hours. Overall, the results suggest that inadequate in-hospital assessment and hasty discharges might be more resource-intensive for healthcare services due to additional work in municipal healthcare services and an increased risk of avoidable hospital readmissions. One might therefore question whether it would be more sustainable for healthcare services if older patients with complex needs were allowed to remain in the hospital for a few extra days to receive a thorough assessment and to facilitate a smooth transition between services.

Research indicates that intermediate wards designated for patients discharged from hospitals might contribute to better discharge processes and collaboration between hospitals and municipal healthcare services [44], as well as reduced length of hospital stay or readmission rates [45]. Participants in our study highlighted that several of the older patients in question received intermediate care in short-term wards in nursing homes or municipal inpatient acute care units. On the other hand, participants working in municipal short-term wards experienced similar challenges related to hasty discharges and insufficient information exchange as those working in home-based services. Based on findings from our study and previous research concerning patient transitions between hospitals and municipal healthcare services [40, 41, 46, 47], we argue that there is a pressing need to enhance integrated care for older frequent users, which includes the development of initiatives and measures aimed at improving cross-sectoral collaboration and routines prior to discharge from hospitals. It is reasonable to suggest that such initiatives could facilitate the determination of adequate follow-up measures and appropriate levels of subsequent healthcare after discharge, ultimately reduce avoidable readmissions.

Like in other countries, Norwegian healthcare policies are based on the ideal of ‘aging in place’ [48]. Our study indicates that significant structural-level challenges exist in providing adequate care to home-dwelling older adults with substantial healthcare needs, particularly those living in rural and remote areas. These challenges include limited access to essential municipal healthcare services, such as round-the-clock home-based care, which could increase the need for hospitalisation. Trends demonstrate that rural areas are more strongly affected by population ageing than urban areas. Simultaneously, there are rural‒urban differences in older people’s health and use of healthcare services, often placing those living in rural areas at a disadvantage [49, 50]. A qualitative interview study involving senior volunteers, family caregivers and healthcare providers of persons with dementia highlighted a mismatch between the perceived needs of care recipients and the extent of home-based care services that could be provided to those living in remote areas of municipalities [51]. A recent national registry study indicated opposite trends, where older adults living in the least central municipalities were more likely to use substantial hospital resources than those living in more central municipalities. However, the study could not determine whether the high use of hospital services among those in rural municipalities was due to insufficient provision of municipal healthcare services [36]. Considering the ‘aging in place’ policies, which apply to everyone regardless of their place of residence, this topic should be explored in future studies.

A previous Norwegian survey study among nurses in hospital and municipal healthcare services indicates that too few older patients were offered intermediate short-term institutional care in the municipalities after discharge from hospital, and the majority reported that they sometimes or often disagreed with transferring older patients to home-based services post-discharge [52]. In our study, several participants perceived it as unsafe for some older frequent users to continue living at home with assistance solely from home-based services. Although these patients could be admitted to municipal intermediate short-term care, typically before admission or after discharge from hospitals, this arrangement was sometimes perceived as insufficient to meet the care recipients’ acute or long-term healthcare needs. At the same time, the threshold for receiving institutional long-term care appeared high due to capacity challenges. This situation can be explained by the fact that while ‘aging in place’ policies have been implemented, there has been a general decline in the proportion of older adults (80+) receiving institutional long-term care [53] along with a reduction in the number of long-term places in Norwegian nursing home [54, 55]. We note that other care arrangements, such as assisted living facilities, were not emphasised in the discussions. Therefore, we cannot determine whether this type of housing was considered suitable for the older adults with substantial healthcare needs addressed in this study. However, these facilities are several places operated by home-based services, which might limit the extent of care provided [51]. In summary, it appears that some older frequent users might ´fall through the cracks`, meaning that, owing to their extensive healthcare needs, it is challenging to find an appropriate level of care for them within municipal healthcare services. Ultimately, this might increase their need for hospital admissions, although hospitalisation might be a short-term solution to patients’ long-term needs.

### Strengths and limitations

Previous research on frequent users of emergency department and hospital services has largely been conducted in hospital settings and by using quantitative study designs [4, 7, 8]. Our study contributes valuable in-depth knowledge to the research field, as municipal healthcare providers meet patients outside the hospital setting and may therefore have complementary experiences and perceptions of care recipients’ healthcare needs. By providing detailed methodological descriptions, we aim to demonstrate transparency in the research process [31]. Additionally, we have included several quotes from the focus group discussions to support our interpretation of the data. However, the study has several limitations that should be acknowledged.

Although the focus group discussions were conducted four years ago (2020–2021), we still consider the data relevant because the organisation of services, including their designated areas of responsibility, remains unchanged. Moreover, no new systems for information exchange between primary and specialist healthcare services have been implemented. We acknowledge that the differing sizes of the focus groups, including two groups with only two participants each–fewer than recommended in the literature [30, 31]–might have affected the data quality. Despite their size, we believe these groups provided valuable insights and diverse perspectives, enriching the overall data with detailed descriptions.

The focus group discussions took place during the COVID-19 pandemic, and it is possible that the participants’ experiences were influenced by the high workload in healthcare services and altered conditions for collaboration between healthcare services and sectors. The pandemic also impacted data collection, with several focus group discussions conducted on a digital platform. We recognise that conducting some focus group discussions in person and others digitally may have influenced participant interactions. However, we did not observe any differences in the richness of the data resulting from this variation.

The study was conducted in a limited number of Norwegian municipalities, and we cannot ignore the possibility healthcare providers elsewhere might have different experiences and perceptions of the healthcare needs of older frequent users and the reasons for their hospital admissions. Questions may also be raised about the fact that the perception of healthcare needs of older frequent users is based on second-hand information from healthcare providers. Future studies should include first-hand perspectives from older frequent users and, in this context, the perspectives of family caregivers. Additionally, the participants in this study were a heterogeneous group comprising healthcare providers with diverse professional backgrounds and from different parts of municipal healthcare services. We argue that this diversity has enriched several of the discussions and may enhance the transferability of the results. Nonetheless, we did not include other essential healthcare providers, such as general practitioners in primary care, or physicians and other healthcare professionals working in hospital settings. Their perspectives should be explored in future research concerning older frequent users.

During the focus group discussions, we did not specifically emphasise avoidable hospital admissions and readmissions. Nonetheless, these concepts have influenced the interpretation of the results and the subsequent discussion. We recommend that future studies explore these issues in greater depth, particularly in relation to frequent users of hospital services. Finally, there is no standard definition or standardised metrics or methods for identifying frequent users, which might result in inconsistent use of the term across studies and study settings [4, 5, 7]. In our study, the participants were not familiar with the term, although they had extensive experience providing care to older patients who were frequently admitted to hospitals. It appears that ´frequent users´ and related terms such as ´high utilisation patients´ are terms adopted in healthcare policies and specialist healthcare services, as well as in research, to draw attention to and investigate patient groups that use significant healthcare resources [5, 32, 33]. To promote integrated care for older adults with complex healthcare needs, we argue that it is important to use concepts and terms that are familiar to healthcare providers across services and sectors.

## Conclusions

International and national health authorities have emphasised the importance of primary care, including municipal healthcare services, in addressing the needs of patients with chronic and long-term conditions and in reducing potentially avoidable unplanned hospital admissions. Older frequent users of somatic hospital services may experience several unplanned admissions, but participants in this study generally did not view these hospitalisations as unnecessary. Instead, they described older patients with severe illnesses and complex healthcare needs that interact with and reinforce each other, with the treatment of acute deterioration often attempted at home or at a higher level of care within the municipality. However, discussions among participants revealed that the reasons for unplanned hospital admissions were not solely due to individual-level factors. Structural-level factors, such as poorly planned and hasty hospital discharges and limited access to adequate healthcare services in municipalities, also contribute. Some unplanned admissions related to structural issues might be avoidable. Based on our findings, we recommend that healthcare policies aimed at reducing unplanned and avoidable hospital admissions address structural-level issues. This includes ensuring access to essential and comprehensive municipal healthcare services and fostering integrated care through collaboration and dialogue between hospitals and municipal healthcare services. For older frequent users of hospital services, addressing individual-level issues, such as severe and complex illnesses and reduced functional abilities, through policy measures is more challenging. Instead of expecting patients to adapt to existing services, policymakers should design services tailored to meet the complex healthcare needs of this patient group.

## Supplementary Information

Supplementary Material 1.

Supplementary Material 2.

## Data Availability

The data material is not publicly available to safeguard participants’ privacy under the General Data Protection Regulation. Anonymised data in Norwegian are available from the corresponding author upon reasonable request.

## Abbreviations

ADLs: Activities of daily living
COPD: Chronic obstructive pulmonary disease
FG: Focus group
HCP: Healthcare provider

## References

[1] OECD. Realising the Potential of Primary Health Care. OECD Health Policy Studies, OECD Publishing, Paris. 2020. Available from: 10.1787/a92adee4-en.

[2] OECD/European Union. Health at a Glance: Europe 2018: State of Health in the EU Cycle. OECD Publishing, Paris. 2018. Available from: 10.1787/health_glance_eur-2018-en.

[3] Brodeur M, Margo-Dermer E, Chouinard M-C, Hudon C. Experience of being a frequent user of primary care and emergency department services: a qualitative systematic review and thematic synthesis. BMJ Open. 2020;10(9):e033351. 10.1136/bmjopen-2019-033351. 32912938 10.1136/bmjopen-2019-033351 PMC7482492

[4] Dufour I, Chouinard M-C, Dubuc N, Beaudin J, Lafontaine S, Hudon C. Factors associated with frequent use of emergency-department services in a geriatric population: a systematic review. BMC Geriatr. 2019;19: 185. 10.1186/s12877-019-1197-9. 31277582 10.1186/s12877-019-1197-9 PMC6610907

[5] Pines JM, Asplin BR, Kaji AH, Lowe RA, Magid DJ, Raven M, et al. Frequent users of emergency department services: gaps in knowledge and a proposed research agenda. Acad Emerg Med. 2011;18(6):e64–9. 10.1111/j.1553-2712.2011.01086.x. 21676051 10.1111/j.1553-2712.2011.01086.x

[6] Ng SH-X, Rahman N, Ang IYH, Sridharan S, Ramachandran S, Wang DD, et al. Characterization of high healthcare utilizer groups using administrative data from an electronic medical record database. BMC Health Serv Res. 2019;19(452). 10.1186/s12913-019-4239-2. 10.1186/s12913-019-4239-2 PMC6612067 31277649

[7] Krieg C, Hudon C, Chouinard M-C, Dufour I. Individual predictors of frequent emergency department use: a scoping review. BMC Health Serv Res. 2016;16:594. 10.1186/s12913-016-1852-1. 27765045 10.1186/s12913-016-1852-1 PMC5072329

[8] Soril LJ, Leggett LE, Lorenzetti DL, Noseworthy TW, Clement FM. Characteristics of frequent users of the emergency department in the general adult population: a systematic review of international healthcare systems. Health Policy. 2016;120(5):452–61. 10.1016/j.healthpol.2016.02.006. 26947060 10.1016/j.healthpol.2016.02.006

[9] Steventon A, Deeny S, Friebel R, Gardner T, Thorlby R. Emergency hospital admissions in England: which may be avoidable and how? London: The Health Foundation. 2018. Available from: https://www.health.org.uk/sites/default/files/Briefing_Emergency%20admissions_web_final.pdf. Accessed 18.12.2024.

[10] Ministry of Health and Care Services. Meld. St. 7 (2019–2020). Nasjonal helse- og sykehusplan 2020–2023. [Report to the Storting NO. 7 (2019–2020). National Health and Hospital Plan 2020–2023]. Oslo: Ministry of Health and Care Services. 2019; Available from: https://www.regjeringen.no/contentassets/95eec808f0434acf942fca449ca35386/no/pdfs/stm201920200007000dddpdfs.pdf. Accessed 18.12.2024.

[11] OECD/European Commission. Health at a Glance: Europe 2024: State of Health in the EU Cycle. OECD Publishing, Paris. 2024. Available from: 10.1787/1ab488a2-en.

[12] OECD. Health at a Glance 2023: OECD Indicators. OECD Publishing, Paris. 2023. Available from: https://doi.org/10.1787/7a7afb35-en.

[13] Conroy T, Heuzenroeder L, Feo R. In-hospital interventions for reducing readmissions to acute care for adults aged 65 and over: an umbrella review. Int J Qual Health Care. 2020;32(7):414–30. 10.1093/intqhc/mzaa064. 32558919 10.1093/intqhc/mzaa064

[14] Lindman AS, Hassani S, Kristoffersen DT, Tomic O, Dimoski T, Helgeland J. 30-dagers overlevelse og reinnleggelse ved norske sykehus for 2013 [30-day survival and readmission at Norwegian hospitals for 2013]. Oslo, Norway: Nasjonalt kunnskapssenter for helsetjenesten; 2014.

[15] Kolk D, Kruiswijk AF, MacNeil-Vroomen JL, Ridderikhof ML, Buurman BM. Older patients’ perspectives on factors contributing to frequent visits to the emergency department: a qualitative interview study. BMC Public Health. 2021. 10.1186/s12889-021-11755-z. 34544405 10.1186/s12889-021-11755-z PMC8454044

[16] Harris LJ, Graetz I, Podila PS, Wan J, Waters TM, Bailey JE. Characteristics of hospital and emergency care super-utilizers with multiple chronic conditions. J Emerg Med. 2016;50(4):e203–14. 10.1016/j.jemermed.2015.09.002. 26472609 10.1016/j.jemermed.2015.09.002

[17] Huang M, Van der Borght C, Leithaus M, Flamaing J, Goderis G. Patients’ perceptions of frequent hospital admissions: a qualitative interview study with older people above 65 years of age. BMC Geriatr. 2020;20: 332. 10.1186/s12877-020-01748-9. 32894056 10.1186/s12877-020-01748-9 PMC7487888

[18] Rosychuk RJ, Chen AA, Ospina MB, McRae AD, Hu XJ, McLane P. Transitions in health care settings for frequent and infrequent users of emergency departments: a population-based retrospective cohort study. BMC Health Serv Res. 2023;23:1250. 10.1186/s12913-023-10260-w. 37964274 10.1186/s12913-023-10260-w PMC10644485

[19] World Health Organization. Integrated care for older people (ICOPE): realigning primary health care to respond to population ageing. Geneva: World Health Organization. 2018. Available from: https://www.who.int/publications/i/item/WHO-HIS-SDS-2018.44. Accessed 12.12.2024.

[20] Saunes IS, Karanikolos M, Sagan A, Norway:Health System Review. Health Systems in Transition. 2020. 22(1); Available from: https://www.who.int/publications/i/item/HiT-22-1-2020. Accessed 14.12.2024. 32863241

[21] Ministry of Health and Care Services. Municipal health and care services. Oslo: Ministry of Health and Care Services. 2022 Available from: https://www.regjeringen.no/no/tema/helse-og-omsorg/helse--og-omsorgstjenester-i-kommunene/omsorgstjenesten/id426407/. Accessed 04.12.2024.

[22] The Norwegian Directorate of Health. Helse-, omsorgs- og rehabiliteringsstatistikk. Eldres helse og bruk av kommunale helse- og omsorgstjenester. [Health-, care- and rehabilitation statistics. Older peoples’ health and the use of municipal health and care services]. Oslo: The Norwegian Directorate of Health; 2016

[23] Ministry of Health and Care Services. Nasjonal helse- og samhandlingsplan 2024–2027. Vår felles helsetjeneste. St. meld. nr. 9 (2023–2024). [Report to the Storting NO. 9 (2023–2024). National Health and Care Coordination Plan 2024–2027. Our Common Healthcare Service]. Oslo: Ministry of Health and Care Services. 2024. https://www.regjeringen.no/no/dokumenter/meld.-st.-9-20232024/id3027594/. Accessed 18.12.2024.

[24] Ministry of Health and Care Services. St.meld.nr.47 (2008–2009) Samhandlingsreformen. Rett behandling - på rett sted - til rett tid. [Report No. 47 to the Storting (2008–2009) The coordination Reform. Proper treatment - at the right place and right time]. Oslo: Ministry of Health and Care Services. 2009: Available from: https://www.regjeringen.no/en/dokumenter/report.no.-47-to-the-storting-2008-2009/id567201/. Accessed 18.12.2024.

[25] Yang F, Burrell LV, Raknes Sogstad MK, Skinner MS. Facility and regional variations in admission and discharge patterns within step-up intermediate care: a cross-sectional study of municipal inpatient acute care services in Norway. Health Serv Insights. 2024. 10.1177/11786329241304565. 39640528 10.1177/11786329241304565 PMC11618911

[26] Steihaug S, Johannessen A-K, Ådnanes M, Paulsen B, Mannion R. Challenges in achieving collaboration in clinical practice: the case of Norwegian health care. Int J Integr Care. 2016;16(3): 3. 10.5334/ijic.2217. 28435416 10.5334/ijic.2217 PMC5351059

[27] Danielsen KK, Nilsen ER, Fredwall TE. Pasientforløp for eldre med kronisk sykdom: En oppsummering av kunnskap. [Patient pathways for older adults with chronic illness: A summary of knowledge]. Norway: Centre for Care Research. 2017. Available from: https://omsorgsforskning.brage.unit.no/omsorgsforskning-xmlui/bitstream/handle/11250/2499035/Danielsen_Nilsen.pdf?sequence=1&isAllowed=y. Accessed 16.12.2024.

[28] Hagen A. Store endringer i kommunekartet- og statistikken. [Major changes in the municipal map and statistics]. 2020. Available from Statistics Norway: https://www.ssb.no/offentlig-sektor/artikler-og-publikasjoner/store-endringer-i-kommunekartet-og-statistikken. Accessed 12.12.2024.

[29] Høydahl, E. Sentralitetsindeksen. Oppdatering med 2020-kommuner. [Centrality Index. Update with 2020-municipalities]. 2020. Available from Statistics Norway: Available from Statistics Norway: https://www.ssb.no/befolkning/folketall/artikler/sentralitetsindeksen . Accessed 12.12.2024.

[30] Polit DF, Beck CT. Nursing research: generating and assessing evidence for nursing practice. 10th ed. Philadelphia, United States: Wolters Kluwer; 2017.

[31] Tong A, Sainsbury P, Craig J. Consolidated criteria for reporting qualitative research (COREQ): a 32-item checklist for interviews and focus groups. Int J Qual Health Care. 2007;19(6):349–57. 10.1093/intqhc/mzm042. 17872937 10.1093/intqhc/mzm042

[32] Myrli TR, Mortensen SM. Eldre stormottakere i somatisk spesialisthelsetjeneste. Analysenotat 05/2018 SAMDATA spesialisthelsetjenesten. [Older high utilization patients in somatic specialist healthcare. Analysis notes 05/2018 SAMDATA specialist healthcare]. Oslo, The Norwegian Directorate of Health. 2018.

[33] Heiberg I. Storforbrukere av somatisk spesialisthelsetjeneste i Helse Nord. SKDE rapport Nr. 3/2015. [High utilization patients in somatic specialist healthcare in Northern Norway. SKDE report NO. 3/2015]. Tromsø, Senter for klinisk dokumentasjon og evaluering. 2015.

[34] Braun V, Clarke V, Hayfield N, Davey L, Jenkinson E. Doing reflexive thematic analysis. In: Bager-Charleson S, McBeath A, editors. Supporting Research in Counselling and Psychotherapy: Qualitative, Quantitative, and Mixed Methods Research: Palgrave Macmillan Cham. 2023:19–38; 10.1007/978-3-031-13942-0

[35] Gjestsen MT, Brønnick K, Testad I. Characteristics and predictors for hospitalizations of home-dwelling older persons receiving community care: a cohort study from Norway. BMC Geriatr. 2018;18: 203. 10.1186/s12877-018-0887-z. 30176794 10.1186/s12877-018-0887-z PMC6122216

[36] Lønhaug-Næss M, Jakobsen MD, Blix BH, Bergmo TS, Hoben M, Moholt J-M. Older high-cost patients in Norwegian somatic hospitals: a register-based study of patient characteristics. BMJ Open. 2023;13:e074411. 10.1136/bmjopen-2023-074411. 37793934 10.1136/bmjopen-2023-074411 PMC10551970

[37] Szilcz M, Wastesson JW, Johnell K, Morin L. Unplanned hospitalisations in older people: illness trajectories in the last year of life. BMJ Supportive Pallitative Care. 2024;14:e953–61. 10.1136/bmjspcare-2020-002778. 10.1136/bmjspcare-2020-002778 33906860

[38] Kröger T. Care poverty: when older people’s needs remain unmet. Palgrave Macmillan Cham. 2022. 10.1007/978-3-030-97243-1.

[39] Ekdahl AW, Hellström I, Andersson L, Friedrichsen M. Too complex and time-consuming to fit in! Physicians’ experiences of elderly patients and their participation in medical decision making: a grounded theory study. BMJ Open. 2012;2(3): e001063. 10.1136/bmjopen-2012-001063. 22654092 10.1136/bmjopen-2012-001063 PMC3367145

[40] Leonardsen A-CL, Werner A, Lurås H, Johannessen A-K. Hospital physicians’ experiences and reflections on their work and role in relation to older patients’ pathways-a qualitative study in two Norwegian hospitals. BMC Health Serv Res. 2022;22: 443. 10.1186/s12913-022-07846-1. 35382820 10.1186/s12913-022-07846-1 PMC8981867

[41] Glette MK, Kringeland T, Røise O, Wiig S. Hospital physicians’ views on discharge and readmission processes: a qualitative study from Norway. BMJ Open. 2019;9:e031297. 10.1136/bmjopen-2019-031297. 10.1136/bmjopen-2019-031297 PMC6720230 31462486

[42] Glans M, Kragh Ekstam A, Jakobsson U, Bondesson Å, Midlöv P. Risk factors for hospital readmission in older adults within 30 days of discharge - a comparative retrospective study. BMC Geriatr. 2020;20:467. 10.1186/s12877-020-01867-3. 33176721 10.1186/s12877-020-01867-3 PMC7659222

[43] van Walraven C, Bell CM. Risk of death or readmission among people discharged from hospital on fridays. Can Med Assoc J. 2002;166(13):1672–3. 12126321 PMC116153

[44] Dahl U, Steinsbekk A, Jenssen S, Johnsen R. Hospital discharge of elderly patients to primary health care, with and without an intermediate care hospital - a qualitative study of health professionals’ experiences. Int J Integr Care. 2014;14: e011. 24868194 10.5334/ijic.1156 PMC4027887

[45] Sezgin D, O’Caoimh R, Liew A, O’Donovan MR, Illario M, Salem MA, et al. The effectiveness of intermediate care including transitional care interventions on function, healthcare utilisation and costs: a scoping review. Eur Geriatr Med. 2020;11(6):961–74. 10.1007/s41999-020-00365-4. 32754841 10.1007/s41999-020-00365-4 PMC7402396

[46] Joo JY, Liu MF. Effectiveness of transitional care interventions for chronic illnesses: a systematic review of reviews. Appl Nurs Res. 2021;61: 151485. 10.1016/j.apnr.2021.151485. 34544575 10.1016/j.apnr.2021.151485

[47] Finlayson K, Chang AM, Courtney MD, Edwards HE, Parker AW, Hamilton K, et al. Transitional care interventions reduce unplanned hospital readmissions in high-risk older adults. BMC Health Serv Res. 2018;18:956. 10.1186/s12913-018-3771-9. 30541530 10.1186/s12913-018-3771-9 PMC6291980

[48] Ministry of Health and Care Services. Meld. St. 24. Fellesskap og meistring-Bu trygt heime. [Report to the Storting NO. 24 (2022–2023). Community and Coping-Live Safely at Home]. Oslo: Ministry of Health and Care Services. 2023. Available from: https://www.regjeringen.no/no/dokumenter/meld.-st.-24-20222023/id2984417/. Accessed: 13.12.2024.

[49] Cohen SA, Greaney ML. Aging in rural communities. Curr Epidemiol Rep. 2023;10:1–16. 10.1007/s40471-022-00313-9. 36404874 10.1007/s40471-022-00313-9 PMC9644394

[50] United Nations Economic Commission for Europe (UNECE). Older persons in rural and remote areas. UNECE Policy Brief on Ageing No. 18. Geneva, Switzerland. UNECE; 2017. Available from: https://www.un-ilibrary.org/content/papers/10.18356/27083047-18/read. UN-iLibrary: Accessed: 02.12.2024.

[51] Blix BH, Hamran T. Assisted living in rural areas: aging in blurred landscapes. Qualitative Res Med Healthc. 2019;3(2). 10.4081/qrmh.2019.7826.

[52] Gautun H, Hartford Kvæl LA, Bratt C. After hospital: should older care-needing patients be transferred to their homes or to an intermediate care institution? Healthcare. 2022;10(3):475. 10.3390/healthcare10030475. 35326952 10.3390/healthcare10030475 PMC8955582

[53] Rostgaard T, Jacobsen F, Kröger T, Peterson E. Revisiting the Nordic long-term care model for older people—still equal? Eur J Ageing. 2022;19:201–10. 10.1007/s10433-022-00703-4. 35528216 10.1007/s10433-022-00703-4 PMC9062627

[54] Statistics Norway, Care. Services 2024; Available from: https://www.ssb.no/en/helse/helsetjenester/statistikk/sjukeheimar-heimetenester-og-andre-omsorgstenester. Accessed 16.12.2024.

[55] Olsen RM, Andfossen NB, Devik SA, Fredwall TE, Førland O, Moholt J-M, Ledelse. pasientsikkerhet og utelatt helse- og omsorgshjelp i sykehjem. Rapport nummer 1/2024. [Leadership, patient safety culture, and missed care in nursing homes. Report NO. 1/2024]. Norway: Centre for Care Research;. 2024. Available from: https://omsorgsforskning.brage.unit.no/omsorgsforskning-xmlui/handle/11250/3128354. Accessed 12.12.2024.

[56] World Medical Association. WMA Declaration Of Helsinki - Ethical Principles for Medical Research Involving Human Participants: World Medical Association. 2025. Available from: https://www.wma.net/policies-post/wma-declaration-of-helsinki/. Accessed: 18.7.2025. 10.1001/jama.2024.21972 39425955

