# Supplementary material for: Municipal healthcare providers’ perceptions of reasons for frequent and unplanned hospital admissions among home-dwelling older adults: a Norwegian focus group study
Source: BMC Geriatr. 2025 Aug 23;25:654. doi: 10.1186/s12877-025-06279-9 (PMC12374330; doi:10.1186/s12877-025-06279-9)
Supplement: Supplementary file 1 — Supplementary Material 1. [file 12877_2025_6279_MOESM1_ESM.docx]

**Supplementary File 1**

**Overview of the focus groups and participants**

| **Focus group** | **Municipality number** | **Healthcare profession** | **Place of employment** | **Identification of participant used in the text** |
| --- | --- | --- | --- | --- |
| FG 1 | 1 | Registered nurse | Home-based services | HCP 1-1 |
| FG 1 | 1 | Licenced practical nurse | Home-based services | HCP 1-2 |
| FG 1 | 1 | Oncology nurse | Home-based services | HCP 1-3 |
| FG 1 | 1 | Registered nurse | Home-based services | HCP 1-4 |
| FG 1 | 1 | Licenced practical nurse | Home-based services | HCP 1-5 |
| FG 2 | 1 | Registered nurse | Home-based services | HCP 2-1 |
| FG 2 | 1 | Licenced practical nurse | Home-based services | HCP 2-2 |
| FG 2 | 1 | Licenced practical nurse | Home-based services | HCP 2-3 |
| FG 2 | 1 | Licenced practical nurse | Home-based services | HCP 2-4 |
| FG 2 | 1 | Registered nurse | Home-based services | HCP 2-5 |
| FG 2 | 1 | Licenced practical nurse | Home-based services | HCP 2-6 |
| FG 3 | 2 | Registered nurse | Short-term ward | HCP 3-1 |
| FG 3 | 2 | Occupational therapist | Day care centre | HCP 3-2 |
| FG 4 | 2 | Physiotherapist | Occupational and physiotherapy unit | HCP 4-1 |
| FG 4 | 2 | Registered nurse | Home-based services | HCP 4-2 |
| FG 5 | 3 | Registered nurse | Home-based services | HCP 5-1 |
| FG 5 | 3 | Registered nurse | Home-based services | HCP 5-2 |
| FG 5 | 3 | Registered nurse | Short-term ward | HCP 5-3 |
| FG 5 | 3 | Registered nurse | Short-term ward | HCP 5-4 |
| FG 5 | 3 | Occupational therapist | Occupational and physiotherapy unit | HCP 5-5 |
| FG 5 | 3 | Physiotherapist | Occupational and physiotherapy unit | HCP 5-6 |
| FG 6 | 4 | Registered nurse | Home-based services | HCP 6-1 |
| FG 6 | 4 | Registered nurse | Home-based services | HCP 6-2 |
| FG 6 | 4 | Registered nurse | Home-based services | HCP 6-3 |
| FG 6 | 4 | Registered nurse | Home-based services | HCP 6-4 |
| FG 6 | 4 | Registered nurse | Home-based services | HCP 6-5 |

FG: focus group; HCP: healthcare provider
