# Supplementary material for: Municipal healthcare providers’ perceptions of reasons for frequent and unplanned hospital admissions among home-dwelling older adults: a Norwegian focus group study
Source: BMC Geriatr. 2025 Aug 23;25:654. doi: 10.1186/s12877-025-06279-9 (PMC12374330; doi:10.1186/s12877-025-06279-9)
Supplement: Supplementary file 2 — Supplementary Material 2. [file 12877_2025_6279_MOESM2_ESM.docx]

**Supplementary File 2**

# Interview guide

**Introductions:** Moderators and participants introduce themselves.

**Study Information:** The moderator provides information about the study and the focus group discussion, emphasising that participation is voluntary, how confidentiality and anonymity will be maintained, and that discussions will be audio-recorded.

**Privacy Reminder**: Participants are reminded not to share personal information about third parties, such as patients and other healthcare personnel.

| **Themes** | **Questions** |
| --- | --- |
| Characteristics of older frequent users of somatic hospital services | The moderator initiates the discussion with a brief description of older frequent users of hospital services, noting that several have complex healthcare needs and experience frequent unplanned admissions (emergency admissions) to somatic hospitals.  Do you provide care to older adults who are frequently and unplanned admitted to somatic hospitals? Please, elaborate.  Based on your experiences, what characterises these patients? (For example, diagnoses, functional abilities, life situation). Please, elaborate and feel free to share anonymised examples. |
| Older frequent users’ use of municipal healthcare services | Could you describe typical municipal healthcare services and follow-up initiatives provided to these patients?  Do you know about older frequent users of hospital services who rarely or never receive municipal healthcare services? Please, elaborate and feel free to share anonymised examples. |
| Prevention of emergency admissions/readmissions | How do you experience the patients’ use of hospital services?  Are there health conditions you are particularly attentive to in terms of avoiding hospital admissions? Please, elaborate.  Can you provide examples of initiatives/measures to avoid admissions?  Is avoidance of hospital admissions/ readmissions a topic among you and your colleagues? Please, elaborate.  Is there any type of expertise you might need related to avoidance of hospital admissions? Please, elaborate.  Do you have examples of factors related to the healthcare services provided to the patients which may influence the frequence of hospital admissions? |
| Collaboration with healthcare providers in hospitals | Can you provide examples of efficient collaboration with healthcare providers in hospitals regarding these patients? What contributed to the well-functioning collaboration?  Do you have examples of collaboration with healthcare providers in hospitals that did not work well? And could the poor quality of the collaboration potentially have led to unnecessary readmissions for these patients? |
| Other aspects | Are there other aspects concerning older adults’ frequent and unplanned hospital admissions that you find important and want to share? |
| Closing | Thank you for participating  Experiences related to participating in the focus group discussion. |
